# Magnonic band spectrum of spin waves in an elliptical helix

**DOI:** 10.1098/rsos.172285

**Published:** 2018-01-31

**Authors:** A. V. Golovchan, V. V. Kruglyak, V. S. Tkachenko, A. N. Kuchko

**Affiliations:** 1 Donetsk National University, Donetsk, Ukraine; 2 Donetsk Institute for Physics and Engineering named after A.A.Galkin, Donetsk, Ukraine; 3 University of Exeter, Exeter, UK; 4 Donetsk National University, Vinnitsa, Ukraine; 5 Institute of Magnetism of NAS of Ukraine, Kiev, Ukraine; 6 Igor Sikorsky Kyiv Polytechnic Institute, Kyiv, Ukraine

**Keywords:** magnonics, spin waves, curvature

## Abstract

We show that the spin-wave spectrum in an elliptical helix has a band character. The size of the first band gap calculated using the perturbation theory is shown to scale as square root of the eccentricity. Curved magnonic waveguides of the kind considered here could be used as structural elements of future three-dimensional magnonic architectures.

## 1. Introduction

The relation between topology and other properties of space forms one of the key aspects in our understanding of nature, including the remarkable connection between the curvature of space and the strength of gravitational field [1]. In particular, it has been shown that curvature plays an important role in physics of low-dimensional systems, where it can be used to alter their electronic and magnetic properties [2–10]. In the context of magnonics (the study of spin waves) and magnonic devices, major research efforts have been devoted to investigation of spin waves [11,12] in curved (or otherwise shaped) magnonic waveguides [13–25]—ubiquitous elements of any magnonic logic architecture [26]—and more generally in non-uniform magnetic configurations [27–38]. The growing variety of proposed magnonic devices and architectures [39–44] ([45] and references therein) [46–48] requires that the nature and diversity of mechanisms of scattering of spin waves in topologically complex magnetic media and graded magnonic landscapes [49–54] be properly understood.

The degree to which spin waves are scattered from a waveguide's bends and the nature of the scattering depends on the character of the spin waves, which in turn depends on the cross-sectional dimensions of the waveguide and the spin-wave frequency and wavelength. The early reports of Bance *et al*. [13] and Dvornik *et al*. [17] suggested minimal scattering of magnetostatic and dipole exchange spin waves from magnonic waveguide bends, which was in contrast to experimental observations of Clausen *et al*. [14] who reported transformation of spin-wave modes propagating along twisted waveguides. The latter experimental results found exhaustive theoretical support in works of Xing *et al*. [18,24], who have offered more detailed numerical simulations of spin wave propagation though bent magnonic waveguides, as compared to [13] and [17]. The role of the applied magnetic field and the internal magnetic field profile was revealed by Sadovnikov *et al*. [55]. Yet, we are still to see a rigorous theory (either analytical or numerical, e.g. based on the procedure devised for monomode waveguides in [43]) of the spin-wave scattering in multimode magnonic waveguides with curved regions. Recently, the spin-wave mode transformation was also observed in T-junctions of magnonic waveguides [56], while the relevance of the graded magnonic index to the spin-wave beam propagation in networks of magnonic waveguides was highlighted in [25].

For exchange spin waves, mostly monomode magnonic waveguides have been considered. No scattering from bends was accounted for (e.g. [57] and references therein), while Tkachenko *et al*. discovered a special kind of geometrical magnetic anisotropy originating from the exchange interaction in curved magnetic nanowires of infinitely small thickness [6,7]. This anisotropy leads to scattering of exchange spin waves from nanowire bends, while a periodic alternation of straight and curved nanowire sections results in formation of a magnonic band spectrum [6,7]. The theory was put on a more rigorous theoretical footing, with a proper account of the effects associated with torsion, by Sheka *et al*. [8,9], while a numerical evidence of the curvature-induced modulation of the exchange field in curved multimode magnonic waveguides was reported in [18].

**Figure 1.**
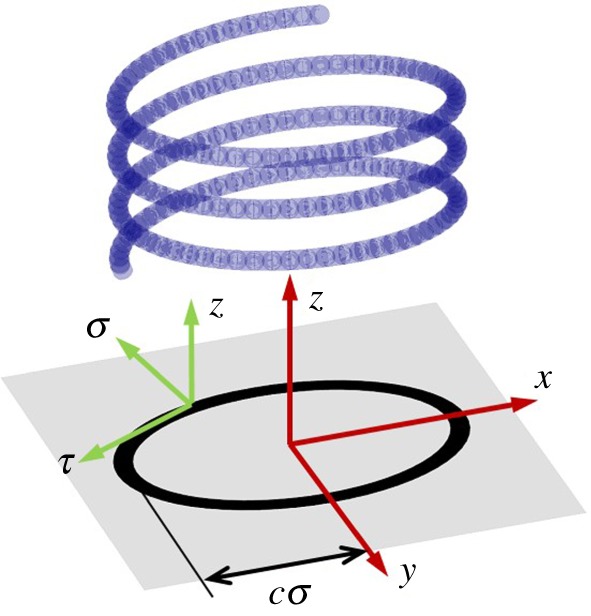
Geometry of the problem and coordinate axes systems used are shown.

Here, we develop a continuous medium theory of the dispersion of exchange spin waves in an ultrathin magnetic nanowire wound so as to form an elliptical helix (figure 1) with an infinitely small pitch. In contrast to a circular helix, the curvature of the elliptical helix is periodically modulated, which in turn modulates the geometrical anisotropy from [6,7]. Using the perturbation theory (similar e.g. to [58]), we show that this modulation leads to a band spectrum for propagating exchange spin waves, with the elliptical helix thereby forming a special kind of a magnonic crystal [59].

## 2. Basic equations

Let us introduce orthogonal curvilinear coordinates of an elliptical cylinder with axes defined by the basis unit vectors **σ**, **τ**, **z** and coordinates *σ* (σ≥1), *τ* (1≥τ≥−1), *z* (figure 1). The magnetic helix is formed by winding a wire around an (imaginary) elliptical cylinder with fixed values of *σ* and parameter *c*, such that *cσ* and 2*c* are equal to the long half-axis and the distance between the foci of the cylinder's elliptical cross-section. We assume the pitch of the helix to be negligible compared to both *cσ* and 2*c*. The turns of the helix are orthogonal to the cylinder's axis. The wire diameter is assumed to be much smaller than the pitch of the helix, so that any interaction between neighbouring turns of the helix could be excluded. The Cartesian coordinates *x*, *y*, *z* are expressed in terms of *σ*, *τ*, *z* as

2.1 $$x=c\sigma \tau ,y^{2}=c^{2}(\sigma^{2}-1)(1-\tau^{2}),\;\text{and}\;z=z.$$

To describe dynamics of the magnetization M(r,t) in the helix, we will use the Landau–Lifshitz equation [11,12]

2.2 $$\frac{\partial \text{M}}{\partial t}=-g[\text{M}\times (\beta (\text{M}\tau )\tau +\alpha \Delta \text{M})],$$

where *β* is the uniaxial anisotropy constant, *α* is the exchange constant, and *g* is the gyromagnetic ratio. There is no external magnetic field applied to the helix. The easy magnetization axis is always directed along the nanowire axis, which coincides with **τ**. Indeed, as shown in [9,60,61], the angle of inclination of the magnetization from the wire direction is proportional to the wire torsion, provided the radius of curvature is greater than the exchange length, which is the case here. The torsion scales with the helix pitch and is therefore approximately equal to zero under our assumptions. Our calculations are performed in the exchange approximation, i.e. neglecting magnetostatic energy (except perhaps its part that could be accounted for within the uniaxial anisotropy constant *β*). This is justified for spin waves with wavelength of the order of the exchange length, which is also the length scale at which the exchange-driven geometrical anisotropy from [6,7] becomes important.

Let us consider small deviations of the magnetization **m**(**r**,*t*) ($|\text{m}|\ll M_{0}$, where *M*_0_ is the saturation magnetization) from the ground state (i.e. the magnetization along the nanowire axis *M*_0_**τ**)

2.3 $$\text{M}(\text{r},t)=M_{0}\tau +\text{m}(\text{r},t),\;{\left[\text{M}(\text{r},t)\right]}^{2}=M_{0}^{2},\;\text{m}\;⊥\;\tau .$$

Linearizing equation (2.2) using **m**(**r**,*t*) as a small parameter, we obtain

2.4 $$\frac{\partial \text{m}}{\partial t}=-g[M_{0}\tau \times \alpha \Delta \text{m}+\text{m}\times \beta M_{0}\tau +\text{m}\times \alpha M_{0}\Delta \tau ].$$

The infinitely small pitch of the helix (and so, the vanishing torsion of the wire) allows us to neglect *z* derivatives in the Laplacian operator in equation (2.4), which therefore becomes [62]

2.5 $$\begin{array}{rl} \Delta \text{m} & =\sigma \frac{1}{c^{2}{\left(\sigma^{2}-\tau^{2}\right)}^{2}}\left[\begin{array}{l} \left(\sigma^{2}-\tau^{2})(1-\tau^{2})\frac{{\text{d}}^{2}}{\text{d}\tau^{2}}m_{\sigma }(\tau \right)\\ \;-\tau (\sigma^{2}-\tau^{2})\frac{\text{d}}{\text{d}\tau }m_{\sigma }(\tau )-(\tau^{2}+\sigma^{2}-1)m_{\sigma }(\tau ) \end{array}\right]\\ & \;+\tau \frac{1}{c^{2}{\left(\sigma^{2}-\tau^{2}\right)}^{2}}\left[2\sigma \sqrt{\left(\sigma^{2}-1)(1-\tau^{2}\right)}\frac{\text{d}}{\text{d}\tau }m_{\sigma }(\tau )-(\tau^{2}+\sigma^{2}-1)M_{0}\right]\\ & \;+\text{z}\frac{1}{c^{2}(\sigma^{2}-\tau^{2})}\left[\left(1-\tau^{2})\frac{{\text{d}}^{2}}{\text{d}\tau^{2}}m_{z}(\tau )-\tau \frac{\text{d}}{\text{d}\tau }m_{z}(\tau \right)\right], \end{array}$$

where *m_σ_*(*τ*) and *m_z_*(*τ*) are the projections of the dynamic magnetization upon the basis vectors **σ** and **z** of the curvilinear coordinate system

2.6 $$\text{m}=m_{\sigma }(\tau )\sigma +m_{z}(\tau )\text{z},$$

and *m_τ_***τ** = 0.

Representing the dynamic magnetization as m(r,t)=m(r)exp⁡{iωt}, substituting τ=cos⁡φ (−∞ < *φ* < +∞) and taking into account equations (2.5) and (2.6), we can write equation (2.4) in the elliptical coordinates as

2.7 $$\begin{array}{rl} & \text{z}:\alpha \frac{{\text{d}}^{2}m_{\sigma }(\varphi )}{\text{d}\varphi^{2}}=\beta c^{2}(\sigma^{2}-\cos^{2}\varphi )m_{\sigma }(\varphi )+i\Omega c^{2}(\sigma^{2}-\cos^{2}\varphi )m_{z}(\varphi )\\ \;\text{and}\; & \sigma :\alpha \frac{{\text{d}}^{2}m_{z}(\varphi )}{\text{d}\varphi^{2}}=\beta c^{2}(\sigma^{2}-\cos^{2}\varphi )m_{z}(\varphi )-\alpha \frac{\left(\sigma^{2}-1+\cos^{2}\varphi \right)}{\left(\sigma^{2}-\cos^{2}\varphi \right)}m_{z}(\varphi )-i\Omega c^{2}(\sigma^{2}-\cos^{2}\varphi )m_{\sigma }(\varphi ), \end{array}\}$$

where $\Omega =\omega /gM_{0}$. The limiting case of a circular helix is obtained from (2.7) by allowing σ→∞,c→0, cσ→ρ, so that

2.8 $$\begin{array}{rl} & \text{z}:i\Omega m_{z}(\varphi )=\frac{\alpha }{\rho^{2}}\frac{{\text{d}}^{2}m_{\rho }(\varphi )}{\text{d}\varphi^{2}}-\beta m_{\rho }(\varphi )\\ \;\text{and}\; & \rho :i\Omega m_{\rho }(\varphi )=-\frac{\alpha }{\rho^{2}}\frac{{\text{d}}^{2}m_{z}(\varphi )}{\text{d}\varphi^{2}}+\left(\beta -\frac{\alpha }{\rho^{2}}\right)\;m_{z}(\varphi ), \end{array}\}$$

which coincides with the corresponding equations from [6].

## 3. Calculation of the magnonic dispersion relation

To calculate the magnonic dispersion relation of spin waves in the nanowire, it is useful to rewrite equations (2.7) in the matrix form

3.1 $$\hat{\text{H}}\mu =0,$$

where the dynamic magnetization (2.6) is written as a two-component column-vector

3.2 $$\mu =\left(\begin{array}{c} m_{\sigma }(\varphi )\\ m_{z}(\varphi ) \end{array}\right)={\left(\begin{array}{cc} m_{\sigma }(\varphi ) & m_{z}(\varphi ) \end{array}\right)}^{T},$$

and the matrix operator $\hat{\text{H}}$ is

3.3 $$\hat{\text{H}}=\left(\begin{array}{cc} \frac{\beta c^{2}}{\alpha }(\sigma^{2}-\cos^{2}\varphi )-\frac{{\text{d}}^{2}}{\text{d}\varphi^{2}} & \frac{i\Omega c^{2}(\sigma^{2}-\cos^{2}\varphi )}{\alpha }\\ -\frac{i\Omega c^{2}(\sigma^{2}-\cos^{2}\varphi )}{\alpha } & \frac{\beta c^{2}}{\alpha }(\sigma^{2}-\cos^{2}\varphi )-\frac{\left(\sigma^{2}-1+\cos^{2}\varphi \right)}{\left(\sigma^{2}-\cos^{2}\varphi \right)}-\frac{{\text{d}}^{2}}{\text{d}\varphi^{2}} \end{array}\right).$$

In view of applying the perturbation theory, we represent the operator (3.3) as a sum of two components

3.4 $$\hat{\text{H}}={\hat{\text{H}}}^{0}+\hat{\text{V}},$$

where ${\hat{\text{H}}}^{0}$ is the operator corresponding to a circular helix

3.5 $${\hat{\text{H}}}^{0}=\left(\begin{array}{cc} \frac{\beta c^{2}\sigma^{2}}{\alpha }-\frac{{\text{d}}^{2}}{\text{d}\varphi^{2}} & \frac{i\Omega c^{2}\sigma^{2}}{\alpha }\\ -\frac{i\Omega c^{2}\sigma^{2}}{\alpha } & \left(\frac{\beta c^{2}\sigma^{2}}{\alpha }-1\right)-\frac{{\text{d}}^{2}}{\text{d}\varphi^{2}} \end{array}\right),$$

and $\hat{\text{V}}$ is a perturbation due to the ellipticity of the helix

3.6 $$\hat{\text{V}}=\frac{c^{2}\cos^{2}\varphi }{\alpha }\;\left(\begin{array}{cc} -\beta & -i\Omega \\ i\Omega & -\beta \end{array}\right)+\left(\begin{array}{cc} 0 & 0\\ 0 & -\frac{2\cos^{2}\varphi -1}{\sigma^{2}-\cos^{2}\varphi } \end{array}\right).$$

Furthermore, instead of parameters *c* and *σ* from (2.1), we introduce parameters *c* and *ρ* that have dimensions of length and are connected via relations

3.7 $$c\sigma =\rho ,\;\left(\sigma =\frac{\rho }{c}\right).$$

Then, introducing notation

3.8 $$\delta \beta =\frac{\alpha }{\rho^{2}},$$

we obtain from (3.5) for ${\hat{\text{H}}}^{0}$

3.9 $${\hat{\text{H}}}^{0}=\left(\begin{array}{cc} \beta -\delta \beta \frac{{\text{d}}^{2}}{\text{d}\varphi^{2}} & i\Omega \\ -i\Omega & (\beta -\delta \beta )-\delta \beta \frac{{\text{d}}^{2}}{\text{d}\varphi^{2}} \end{array}\right),$$

and from (3.6) for $\hat{\text{V}}$

3.10 $$\hat{\text{V}}=E\cos^{2}\varphi \;\left(\begin{array}{cc} -\beta & -i\Omega \\ i\Omega & -\beta \end{array}\right)+\delta \beta \;\left(\begin{array}{cc} 0 & 0\\ 0 & 1-\frac{1-E\sin^{2}\varphi }{1-E\cos^{2}\varphi } \end{array}\right),$$

where $E=c^{2}\text{/}\rho^{2}=1\text{/}\sigma^{2}$.

As mentioned earlier, we aim to derive the spectrum of spin waves in an elliptical helix considering its eccentricity as a perturbation relative to a circular helix. In this approach, quantity ε=E/2≪1 is considered as a small parameter of the perturbation theory. The problem can then be reformulated as one of finding the spectrum of equation (3.1) with $\hat{\text{H}}$ given by

3.11 $$\hat{\text{H}}={\hat{\text{H}}}^{0}-\varepsilon \hat{{\text{V}}^{\prime }},$$

where with notations

3.12 $$K=\frac{\beta }{\delta \beta },\;W=\frac{\Omega }{\delta \beta }$$

${\hat{\text{H}}}^{0}$ has form

3.13 $${\hat{\text{H}}}^{0}=\left(\begin{array}{cc} K-\frac{{\text{d}}^{2}}{\text{d}\varphi^{2}} & iW\\ -iW & (K-1)-\frac{{\text{d}}^{2}}{\text{d}\varphi^{2}} \end{array}\right),$$

and the perturbation operator in linear in *ϵ* approximation is obtained from (3.10) as

3.14 $$\hat{{\text{V}}^{\prime }}={\hat{\text{V}}}_{0}+{\hat{\text{V}}}_{1}\cos(2\varphi ),\;\text{where}\;{\hat{\text{V}}}_{0}=\left(\begin{array}{cc} K & iW\\ -iW & K \end{array}\right)\;\text{and}\;{\hat{\text{V}}}_{1}=\left(\begin{array}{cc} K & iW\\ -iW & K+2 \end{array}\right).$$

The eigenfunctions of ${\hat{\text{H}}}^{0}$ have the form of plane waves

3.15 $$\mu^{\left(0\right)}=\left(\begin{array}{c} m_{\sigma }^{\left(0\right)}\\ m_{z}^{\left(0\right)} \end{array}\right)\;\exp\{ik\varphi \},$$

where $m_{\sigma }^{\left(0\right)}$ and $m_{z}^{\left(0\right)}$ are wave amplitudes that define the spin-wave polarization. The spectrum of the spin waves is

3.16 $$W^{2}=(K+k^{2})\;(K-1+k^{2}),$$

which coincides with the corresponding results from [6,7].

Owing to the periodicity of the perturbation operator (the period of which is π), the spectrum and eigenfunctions of the problem are also periodic. Hence, let us introduce a one-dimensional reciprocal lattice $k_{n}=2n,\;\;n$ is integer, so that the boundaries of the first Brillouin zone correspond to Q=±1. The following calculation is then performed using the standard perturbation theory from [63].

The states near the centre (small *k* values) and boundaries (k≈Q) of the Brillouin zone are affected by the perturbation (3.14) differently. In the former case (Brillouin zone centre), the main contribution is due to the constant term in (3.14), in which case we obtain from equation (3.16)

3.16a $$W^{2}{\left(1-\varepsilon \right)}^{2}=(K(1-\varepsilon )+k^{2})\;(K(1-\varepsilon )-1+k^{2})$$

or

3.16b $$W^{2}\approx (K+k^{2})\;(K+k^{2}-1)+\varepsilon \;\left(2k^{2}(K+k^{2}-1)-\frac{K}{2}\right).$$

For the case of k≈Q, the eigenfunctions of operator $\hat{\text{H}}$ given by (3.11) (and therefore the solutions of equation (3.1)) can be found via expansion in terms of eigenfunctions of the unperturbed operator ${\hat{\text{H}}}^{0}$ given by (3.13)

3.17 $$\mu =\left(\begin{array}{c} m_{\sigma }^{\left(k\right)}\\ m_{z}^{\left(k\right)} \end{array}\right)\;\exp\{ik\varphi \}+\left(\begin{array}{c} m_{\sigma }^{\left(k+q\right)}\\ m_{z}^{\left(k+q\right)} \end{array}\right)\;\exp\{i(k+q)\varphi \},$$

where *q* = −2 is a reciprocal lattice vector. Following [63], we substitute expansion (3.17) into equation (3.1), multiply the result by exp⁡{−ikφ} and then by exp⁡{−i(k+q)φ}, and act on the result by integral operator $1\text{/}\pi \int_{0}^{\pi }\ldots \text{d}\varphi$, to obtain the following system of equations

3.18 $$\left\{ \begin{array}{l} \frac{1}{\pi }\int_{0}^{\pi }{\text{e}}^{-ik\varphi }({\hat{\text{H}}}^{0}-\varepsilon \hat{{\text{V}}^{\prime }}){\text{e}}^{ik\varphi }\text{d}\varphi \cdot \left(\begin{array}{c} m_{\sigma }^{\left(k\right)}\\ m_{z}^{\left(k\right)} \end{array}\right)+\int_{0}^{\pi }{\text{e}}^{-ik\varphi }({\hat{\text{H}}}^{0}-\varepsilon \hat{{\text{V}}^{\prime }}){\text{e}}^{i(k+q)\varphi }\text{d}\varphi \cdot \left(\begin{array}{c} m_{\sigma }^{\left(k+q\right)}\\ m_{z}^{\left(k+q\right)} \end{array}\right)=0\\ \frac{1}{\pi }\int_{0}^{\pi }{\text{e}}^{-i(k+q)\varphi }({\hat{\text{H}}}^{0}-\varepsilon \hat{{\text{V}}^{\prime }}){\text{e}}^{ik\varphi }\text{d}\varphi \cdot \left(\begin{array}{c} m_{\sigma }^{\left(k\right)}\\ m_{z}^{\left(k\right)} \end{array}\right)+\int_{0}^{\pi }{\text{e}}^{-i(k+q)\varphi }({\hat{\text{H}}}^{0}-\varepsilon \hat{{\text{V}}^{\prime }}){\text{e}}^{i(k+q)\varphi }\text{d}\varphi \cdot \left(\begin{array}{c} m_{\sigma }^{\left(k+q\right)}\\ m_{z}^{\left(k+q\right)} \end{array}\right)=0. \end{array}\right.$$

This is a homogeneous system of linear (with respect to spin-wave amplitudes $m_{\sigma }^{\left(k\right)}$, $m_{z}^{\left(k\right)}$, $m_{\sigma }^{\left(k+q\right)}$, $m_{z}^{\left(k+q\right)}$) equations that can be written in matrix form as

3.19 $$\hat{T}\;\left(\begin{array}{c} m_{\sigma }^{\left(k\right)}\\ m_{z}^{\left(k\right)}\\ m_{\sigma }^{\left(k+q\right)}\\ m_{z}^{\left(k+q\right)} \end{array}\right)=0.$$

This system has non-trivial solutions if and only if its determinant is equal to zero

3.20 $$\det\;\hat{T}=0.$$

Taking into account that the perturbation potential (3.14) is a periodic function with period π, solutions of this equation for *k* = 1 (i.e. for the Brillouin zone boundary) and *q* = −2 (reciprocal lattice vector) will define the frequency boundaries *W*_±_ and size $\Delta W=W_{+}-W_{-}$ of the magnonic band gap in the spin-wave spectrum.

So, introducing notation

3.21 $$\tilde{\Omega }=\frac{W^{2}}{K(K+1)}$$

equation (3.20) becomes

3.22 $${\tilde{\Omega }}^{2}-2B\tilde{\Omega }+C=0,$$

where the coefficients in linear in *ϵ* approximation are B≈1+(ε/(K+1)), C≈1−(2Kε/(K+1)).

Solutions of equation (3.22) in the lowest order in *ε* are

3.23 $${\tilde{\Omega }}_{\pm }\approx 1\pm \sqrt{2\varepsilon }.$$

So, we finally obtain for the boundaries of the band gap

3.24 $$W_{\pm }\approx \sqrt{K(K+1)}\left\lfloor 1\pm \sqrt{\frac{\varepsilon }{2}}\right\rfloor ,$$

and for the size of the magnonic band gap

3.25 $$\Delta \Omega =\sqrt{\beta (\beta +\delta \beta )}\sqrt{2\varepsilon },$$

or taking into account notations introduced earlier in (2.7), (3.7), (2.8), (3.10),

3.26 $$\Delta \omega =gM_{0}\frac{c}{\rho }\sqrt{\beta \left(\beta +\frac{\alpha }{\rho^{2}}\right)}.$$

## 4. Discussion

Owing to technological reasons, the radius of curvature of magnetic nanowires can hardly be made comparable or smaller than the exchange length, which is of the order of 10 nm for most popular magnonic materials, such as permalloy or yttrium-iron garnet (YIG). Moreover, the radius of curvature is actually required to be greater than the exchange length to ensure stability of the magnetization along the wire length [6,8,9]. So the curvature-induced anisotropy should always be *δβ* = *α*/*ρ*^2^ < 1. For the sake of an estimate, we can take the uniaxial anisotropy strength to be *β* ≈ 2π, i.e. about the strength of the shape anisotropy in a straight nanowire. Thus, we can see that *δβ* should generally be expected to be much smaller than *β*. Then, equation (3.26) allows us to estimate the band gap size as

4.1 $$\Delta \omega =\frac{c}{\rho }\omega_{0},$$

where *ω*_0_ = *βgM*_0_ is the frequency of the uniform ferromagnetic resonance in a straight nanowire. Remarkably, this result does not depend on the exchange parameter but is only determined by the aspect ratio of the helix. This suggests that the band gap is of topological origin, which is similar to the topological modulation of the dispersion of a quantum-mechanical electron moving along a curved path [3–5].

The size of the first allowed magnonic band can be estimated as

4.2 $$\omega_{1}=\omega_{0}\left(\sqrt{1+\frac{\delta \beta }{\beta }}-\sqrt{1-\frac{\delta \beta }{\beta }}\right)\approx \frac{\alpha }{\rho^{2}}\omega_{0},$$

which depends upon both the exchange parameter and the curvature of the nanowire. The ratio of the first band gap to the first allowed band sizes is

4.3 $$\frac{\Delta \omega }{\omega_{1}}\approx \frac{c\rho }{\alpha }.$$

The width ratio of the magnonic bands and band gaps is a key characteristic of magnonic crystals [64–66]. Equations (4.1–4.3) demonstrate that the magnonic band gap spectrum of elliptical helices studied here can be tailored within a wide range, in accordance with conclusions of [6]. Both *c* and *ρ* either should or can easily be imagined to exceed the exchange length. For the sake of an estimate, one could take 20 nm, 100 nm, 10 GHz and 100 nm^2^ for *c*, *ρ*, *ω*_0_/2π, and *α*, respectively. This would yield 2 and 0.1 GHz for the first magnonic band gap and allowed band, respectively. The essentially flat first allowed band is due to the relatively small group velocity of exchange spin waves of long wavelength, pointing to the lack of the magneto-dipole field in our formalism.

In terms of experimental observation of the peculiar magnonic spectrum described above, one would need to overcome the following two major obstacles. Firstly, the helix needs to be fabricated from a magnetic material in which the spin-wave propagation length would exceed the length of a few turns of the helix [64,66]. Secondly, the effects could be masked by those due to the inherent non-uniformity of the micromagnetic configurations and associated magnonic index in realistic samples [25,55,67,68]. The experimental challenges are, however, common for the entire field of nano-magnonics [69] and will hopefully be overcome eventually. On the theoretical side, it would be interesting to generalize the calculations to the case of dipole-exchange spin waves [12] and to include the effects associated with the torsion, e.g. following the approach laid out in [8,9,60,61,70].

## 5. Conclusion

In summary, we have shown that the spin-wave spectrum of an elliptical helix is characterized by the presence of a magnonic band gap. The size of the band gap has been calculated using the perturbation theory and shown to scale as the square root of the eccentricity, or the ratio of the inter-foci distance to the long half-axis of the ellipses forming the helix. Curved magnonic waveguides of the kind considered here could be used as structural elements of future three-dimensional magnonic architectures.

## Data accessibility

All data related to this research are contained in the manuscript.
